# GSE is now an open access journal published by BioMed Central

**DOI:** 10.1186/1297-9686-41-1

**Published:** 2009-01-05

**Authors:** Hélène Hayes, Philippe Baret, Didier Boichard

**Affiliations:** 1 INRA, UMR de Génétique Animale et Biologie Intégrative, Jouy-en-Josas, France; 2 Université Catholique de Louvain, Unité de Génétique, Louvain-La-Neuve, Belgium; 3 INRA, Département de Génétique Animale, Jouy-en-Josas, France

It is our pleasure to welcome you to the new website of *Genetics Selection Evolution *(*GSE*).

From January 2009, *GSE *is the first "Open Access" (OA) journal dedicated to original research on genetics and selection in farm and experimental animals. Converting *GSE *to OA provides an online journal freely accessible to all readers without charge, and is an opportunity we did not want to miss. This has already been largely explained in our previous editorial [1].

In addition, we are really pleased to announce that BioMed Central (BMC) will publish *GSE *from January 2009. BMC is the largest publisher of peer-reviewed open access journals in biology and medicine. BMC's journals fall into two main groups *i.e*. (1) the BMC series journals and (2) the independent journals with their own editorial control, which now includes *GSE*. Thus, *GSE *remains the full property of INRA and keeps its name, scope and editorial policy.

BMC offers high-quality technology for editorial tools and functionalities that enable effective communication of the scientific work. All articles in BMC journals are published in HTML and PDF (searchable and offering colour) versions and are also made available in machine-readable XML format. *GSE *articles will be deposited in PubMed Central as well as in other freely accessible archives and repositories .

Back issues of *GSE *and of its parent journal (Annales de Génétique et de Sélection Animales) from 1969 till 2008 will soon be relocated to the new site but remain available online at EDP Sciences  and free of charge. We take this opportunity to thank EDP Sciences, the publisher of *GSE *from 2000 to 2008, for the excellent service they have provided.

We are also grateful to the people at the Business Development Department of INRA for their continued support during this important time of change.

Now we invite you to discover the first articles released in this new format, with many more to come, that we believe reflect well the range of topics covered by *GSE*.
